# Momast Protects SH-SY5Y Cells Against Heavy Metal-Induced Neurotoxicity Through Complementary Membrane-Dependent and Intracellular Mechanisms

**DOI:** 10.3390/ijms27188157

**Published:** 2026-09-13

**Authors:** Daniela Meleleo, Alexia Barbarossa, Roberta Tardugno, Angelica Spano, Alessia Carocci, Maria Lisa Clodoveo, Filomena Corbo, Rosanna Mallamaci

**Affiliations:** 1 Department of Science of Agriculture, Food, Natural Resources and Engineering, University of Foggia, 71122 Foggia, Italy; daniela.meleleo@unifg.it; 2 Department of Pharmacy-Pharmaceutical Sciences, University of Bari Aldo Moro, 70125 Bari, Italy; alexia.barbarossa@uniba.it (A.B.); alessia.carocci@uniba.it (A.C.); filomena.corbo@uniba.it (F.C.); 3 Interdisciplinary Department of Medicine, University of Bari Aldo Moro, 70124 Bari, Italy; a.spano8@phd.uniba.it (A.S.); marialisa.clodoveo@uniba.it (M.L.C.); 4 Department of Biosciences, Biotechnologies and Environment, University of Bari Aldo Moro, 70125 Bari, Italy

**Keywords:** heavy metals, Momast, neuroprotective effect, planar lipid membrane

## Abstract

Momast is a patented polyphenolic complex sustainably extracted from the vegetation water generated during the processing of the Coratina olive cultivar. Although its antioxidant and anti-inflammatory properties have been previously demonstrated, its neuroprotective activity and underlying mechanisms remain largely unexplored. In this study, we investigated the neuroprotective effects of Momast using complementary cellular and biophysical approaches. SH-SY5Y human neuroblastoma cells were exposed to cadmium, mercury, or lead to reproduce distinct mechanisms of heavy metal-induced neurotoxicity, and cell viability was evaluated following co-treatment with Momast. In parallel, the interaction of Momast with biomimetic planar lipid membranes composed of phosphatidylcholine and cholesterol was investigated by electrophysiological analysis. Momast significantly attenuated heavy metal-induced cytotoxicity, although the magnitude and concentration dependence of the protective effect varied according to the metal. Electrophysiological measurements demonstrated that Momast interacts directly with lipid bilayers through a sequential adsorption–insertion process, without compromising membrane integrity, and forms stable, conductive assemblies within the membrane. Overall, the combined cellular and biophysical findings support a mechanistic model in which Momast exerts neuroprotective activity through complementary membrane-dependent and intracellular mechanisms, highlighting membrane interactions as an important determinant of the biological activity of complex olive polyphenolic mixture.

## 1. Introduction

Extra virgin olive oil is a major component of the Mediterranean diet and an important dietary source of bioactive phenolic compounds. Its beneficial health effects have been associated not only with its high content of monounsaturated fatty acids but also with its complex mixture of minor constituents, including phenolic compounds [1,2,3]. Olive-derived polyphenols, such as hydroxytyrosol, tyrosol, oleuropein, oleacein, oleocanthal, and verbascoside, exhibit a broad range of biological activities, including antioxidant and anti-inflammatory effects [4,5,6]. Importantly, increasing evidence suggests that the biological activity of olive polyphenols may result from interactions among multiple constituents rather than from the activity of individual compounds, supporting the investigation of complex polyphenolic mixtures [7].

Over the last decade, considerable efforts have been devoted to elucidating the molecular mechanisms underlying the biological activities of olive polyphenols. In addition to their well-established radical-scavenging activity, these compounds regulate multiple intracellular signalling pathways involved in oxidative stress, inflammation, apoptosis, mitochondrial function, and lipid metabolism. Furthermore, increasing evidence indicates that their biological effects also depend on their ability to interact with biological membranes, which function not only as structural barriers but also as dynamic platforms regulating cell signalling and homeostasis [8].

Olive mill by-products, including vegetation water generated during olive oil production, represent valuable sources of phenolic compounds and have attracted increasing interest as sustainable raw materials within the circular economy [9,10,11,12].

Momast is a patented polyphenolic complex obtained from the vegetative water of the Coratina olive cultivar through physical and mechanical extraction procedures. Its chemical characterization has demonstrated the presence of several phenolic compounds, including hydroxytyrosol, tyrosol, oleuropein, and verbascoside [13,14,15].

The unique phytochemical profile of Momast has prompted extensive investigation of its biological activities. Previous studies have demonstrated its antioxidant and anti-inflammatory activities, together with its ability to modulate intestinal contractility, suggesting potential applications in gastrointestinal disorders [15]. Additional evidence has shown that Momast reduces reactive oxygen species production, downregulates inducible nitric oxide synthase expression, and exerts cardioprotective effects [16]. Moreover, it improves cholesterol homeostasis and attenuates oxidative stress both in HepG2 cells [13] and in hypercholesterolemic mouse models [14]. More recently, Momast has also been shown to regulate Aquaporin-3 expression and epithelial–mesenchymal transition markers in colorectal cancer cells, indicating that its biological activity extends beyond its antioxidant properties and involves the modulation of intracellular signalling pathways controlling cell survival and proliferation [17].

Although the neuroprotective potential of olive polyphenols and olive mill wastewater extracts has been extensively documented [18,19,20,21,22], the effects of Momast on neuronal cells remain largely unexplored. In particular, whether direct interactions between Momast and biological membranes contribute to its cellular activity has not yet been established. This question is relevant because the plasma membrane represents the first interface between cells and bioactive compounds and plays a critical role in regulating membrane integrity, permeability, redox signaling, and cellular homeostasis.

Based on these considerations, we hypothesized that the neuroprotective effects of Momast arise from complementary membrane-dependent and intracellular mechanisms. To test this hypothesis, we combined two complementary experimental approaches. First, we evaluated the ability of Momast to protect SH-SY5Y human neuroblastoma cells from heavy metal-induced neurotoxicity. Cadmium (Cd), Mercury (Hg), and Lead (Pb) were selected because they reproduce distinct yet complementary pathogenic mechanisms involved in neurodegeneration, including oxidative stress, mitochondrial dysfunction, calcium dyshomeostasis, neuroinflammation, and neuronal injury [23,24]. Second, we investigated the interaction of Momast with planar lipid membranes (PLMs) composed of phosphatidylcholine and cholesterol (POPC = 65:35, *w*/*w*), a well-established biomimetic model that reproduces the lipid composition of the outer leaflet of aged neuronal plasma membranes [25]. Among the available membrane models, PLMs provide a powerful platform for investigating the direct interaction of bioactive molecules with lipid bilayers under controlled experimental conditions [26,27,28]. Electrophysiological measurements were used to assess Momast-induced changes in membrane capacitance and conductance, as well as the formation and properties of conductive structures. By integrating cell biology and membrane biophysics, this study provides a mechanistic framework that links the membrane-interacting properties of Momast to its neuroprotective activity against heavy metal-induced neuronal injury.

## 2. Results

### 2.1. Characterization of Momast: Total Phenolic Content and Antioxidant Activity

The total phenolic content (TPC) and total antioxidant activity (TAA) of Momast were evaluated using complementary colorimetric assays. The results are summarized in Table 1. TPC was determined using both the Folin–Ciocalteu (F–C) and Fast Blue BB (FBBB) assays and expressed as gallic acid equivalents (GAE), whereas antioxidant activity was assessed by the ABTS, DPPH, and FRAP assays and expressed as Trolox equivalents (TE).

The TPC values obtained with the two methods differed markedly. The Folin–Ciocalteu assay yielded a value of 209.07 mg GAE/mL, whereas the Fast Blue BB assay gave a substantially lower value (50.29 mg GAE/mL), corresponding to an F–C/FBBB ratio of 4.15.

The antioxidant activity measured by the ABTS and DPPH assays was comparable, whereas the FRAP assay produced considerably higher values (Table 1).

The TPC determined for the Momast sample analyzed in the present study was higher than those previously reported for commercially available Momast formulations, including HY100, PLUS30, PW25, and MP30B [15,16].

### 2.2. Effect of Cd, Hg, Pb or Momast on Cells

**Figure 1:**
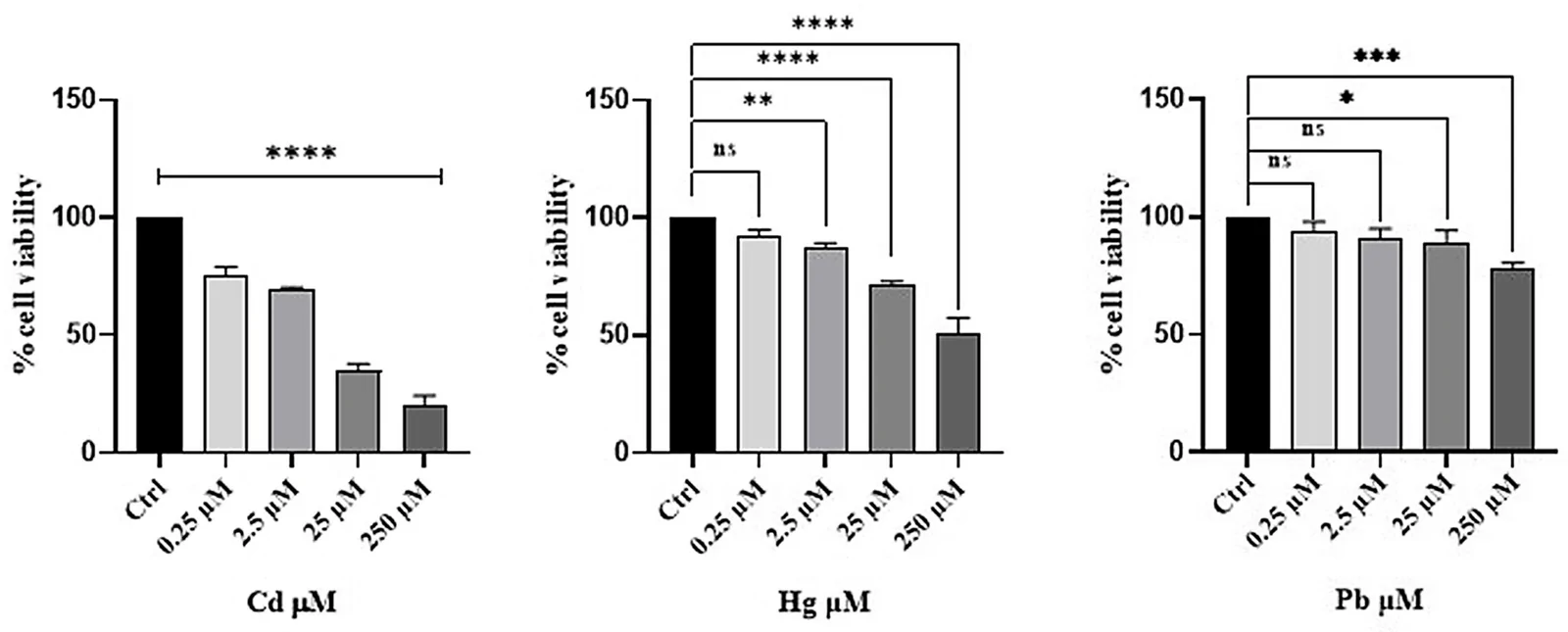
Effect of CdCl_2_, HgCl_2_, and PbCl_2_ on SH-SY5Y cell viability after 24 h of exposure (MTT assay). Data are expressed as a percentage of vehicle-treated cells (control). Results are shown as mean ± standard deviation (SD) (n = 3). Non-significant differences (ns, *p* > 0.05), * *p* < 0.05, ** *p* < 0.01, *** *p* < 0.001, and **** *p* < 0.0001 as compared with the control.

To evaluate the cytotoxic effects of heavy metal exposure, cell viability was assessed following treatment for 24 h with increasing concentrations (0.25, 2.5, 25, and 250 µM) of Cd, Hg, or Pb on SH-SY5Y cells relative to an untreated control. The concentration range of CdCl_2_, HgCl_2_, and PbCl_2_ used in the present study (0.25–25 μM) was selected to encompass low-to-moderate exposure levels reported in experimental studies, whereas 250 μM was included as a high-concentration condition to induce measurable acute cytotoxicity. These concentrations should be interpreted as in vitro exposure concentrations rather than as chronic or acute doses in humans or animals [29,30,31,32,33,34]. All three heavy metals induced a decrease in cell viability, though with distinct toxicity profiles. Cd exhibited the highest cytotoxicity among the tested metals. A significant reduction in cell viability of approximately 25%, 30%, 65% and 80% was observed at concentrations of 0.25, 2.5, 25, and 250 µM, respectively. Treatment with Hg also reduced cell viability in a dose-dependent manner, though the effect was less severe than that of cadmium. The minor decrease observed at lower concentration, 0.25 µM (7% reduction), did not reach statistical significance compared to the control. However, significant cytotoxicity was observed at 2.5 µM with a 13% decrease, and became increasingly severe at higher concentrations, showing a 30% and 50% reduction at 25 and 250 µM, respectively. Similarly, Pb demonstrated the lowest relative toxicity. Low-dose exposures showed no significant difference from the control group, resulting in minor decreases of 6% a 0.25 µM and 9% at 2.5 µM. A statistically significant drop was observed at 25 µM, with an 11% reduction in cell viability, and became more pronounced at 250 µM, leading to a maximum reduction of approximately 22% (Figure 1). The corresponding MTT dose–response curves and dot plots showing the individual data points are provided in the Supporting Information (Figures S1–S7).

**Figure 2:**
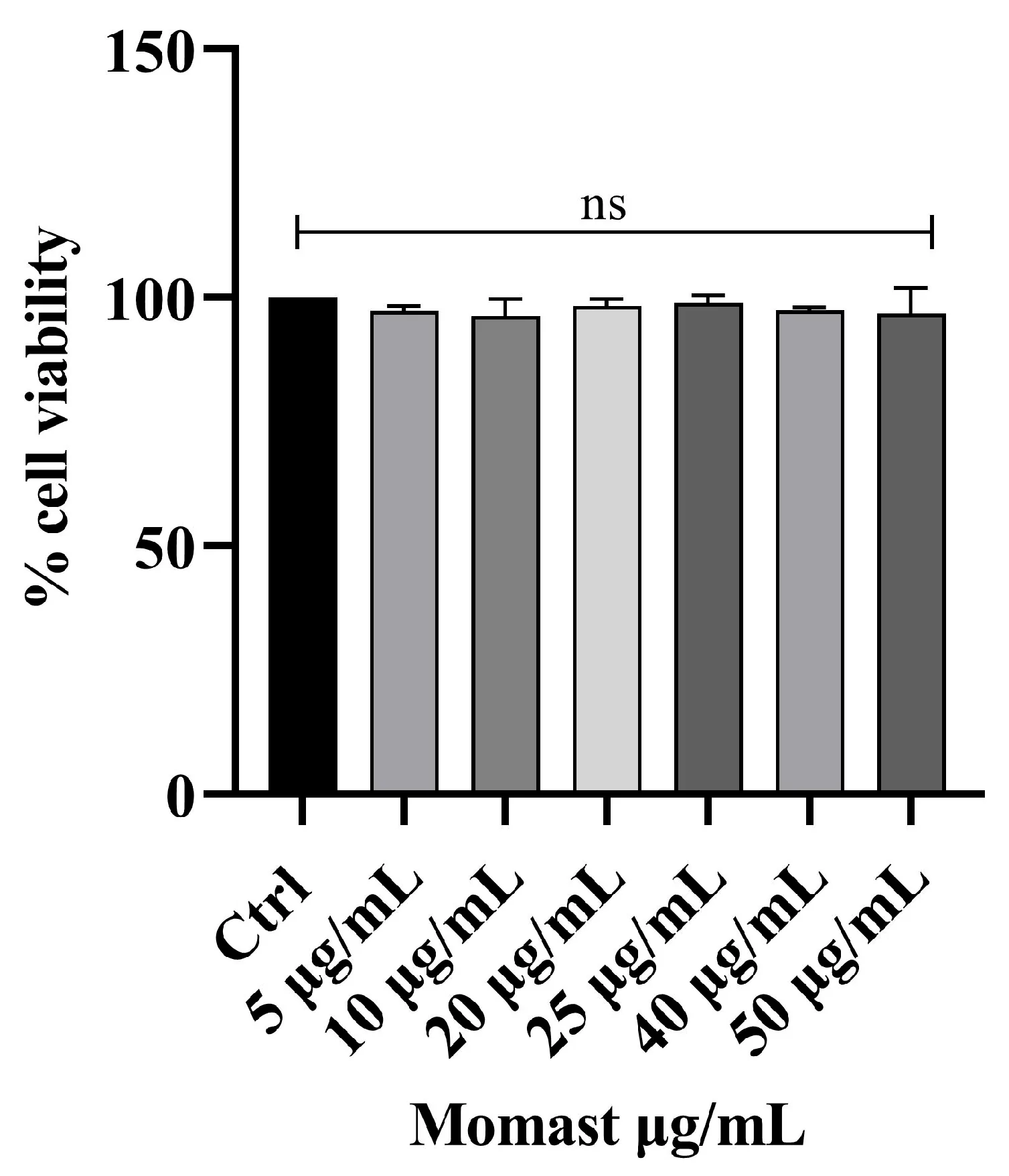
Effect of Momast on SH-SY5Y cell viability after 24 h of exposure (MTT assay). Data are expressed as a percentage of vehicle-treated cells (control). Results are shown as mean ± standard deviation (SD) (n = 3). No statistically significant differences were observed compared with the control (ns, *p* > 0.05).

Subsequently, the effects of Momast on cell viability were evaluated within a 5–50 µg/mL concentration range for 24 h. Momast did not significantly affect cell viability at any concentration tested (5–50 μg/mL), indicating no detectable cytotoxicity under the present experimental conditions (Figure 2). This result indicates that Momast did not exert detectable cytotoxic effects under the experimental conditions investigated.

### 2.3. Cytoprotective Effects of Momast Against Heavy Metal-Induced Toxicity

To investigate the potential neuroprotective effects of the polyphenolic complex, SH-SY5Y cells were co-treated with Cd, Hg, or Pb in combination with increasing concentrations of Momast. Based on our preliminary findings and in line with our previous investigations [35], a concentration of 25 μM for the heavy metals and doses of 5, 25, and 50 µg/mL for Momast were selected for the subsequent cytoprotection assays. The 25 μM metal concentration was chosen because it consistently induces significant cytotoxicity while maintaining enough viable cells to test potential protective agents. Concurrently, the selected Momast concentrations correspond to the minimum, intermediate, and maximum effective doses established during the initial biocompatibility screening.

**Figure 3:**
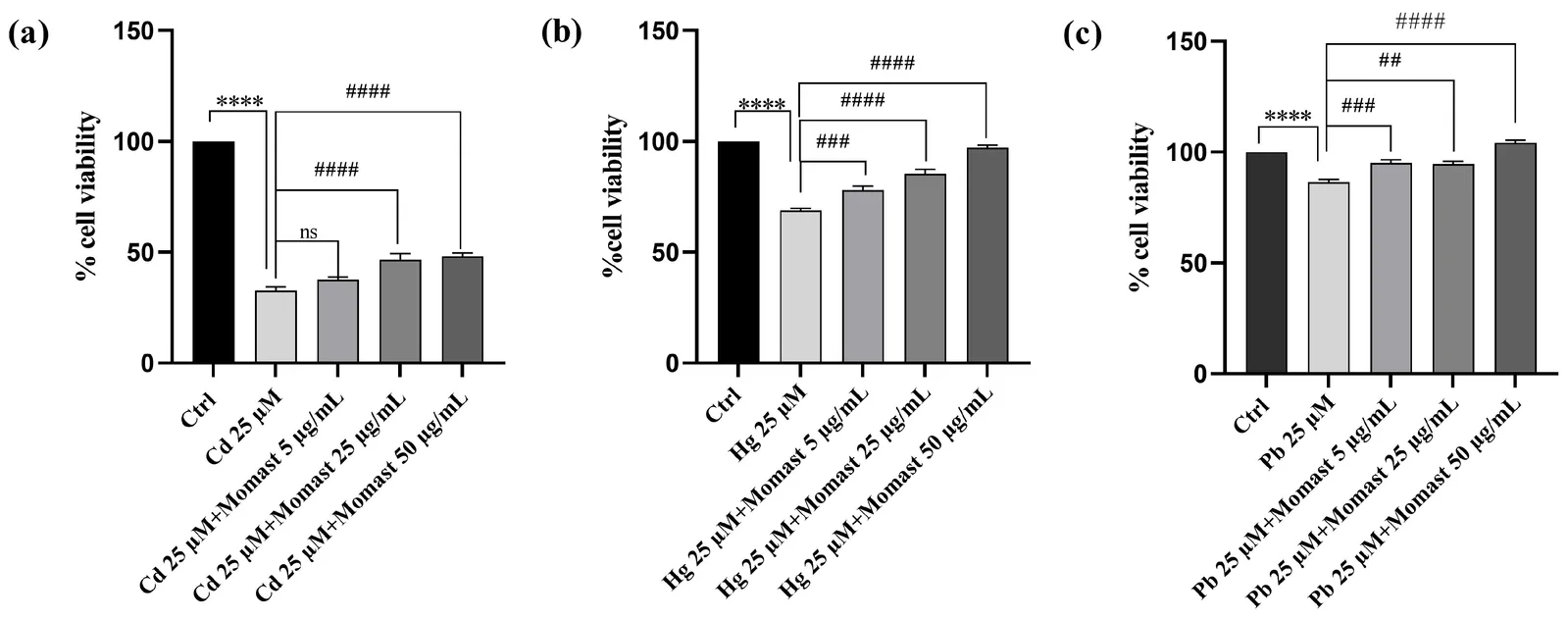
Effect of simultaneous treatment with CdCl_2_ (**a**), HgCl_2_ (**b**), PbCl_2_ (**c**), and MOMAST on SH-SY5Y cell viability after 24 h of exposure (MTT assay). Data are expressed as mean ± standard deviation (SD) (n = 3). Significant differences versus control **** *p* < 0.0001. Significant differences were in: (**a**) non-significant differences (ns, *p* > 0.05), #### *p* < 0.0001 as compared to CdCl_2_; (**b**) ### *p* < 0.05, #### *p* < 0.0001 as compared to HgCl_2_; (**c**) ## *p* < 0.01, ### *p* < 0.05, #### *p* < 0.0001 as compared to PbCl_2_.

Exposure to 25 μM Cd reduced cell viability to approximately 35% of the control value. Co-treatment with Momast at 5 μg/mL did not significantly modify Cd-induced cytotoxicity. In contrast, 25 and 50 μg/mL Momast significantly increased cell viability to approximately 45% and 47%, respectively, compared with Cd-treated cells (Figure 3a).

Treatment with 25 μM Hg caused a significant decrease in cell viability, dropping to approximately 71% compared to the untreated control. Momast displayed a clear, concentration-dependent protective profile against mercury-induced toxicity. When compared to the group treated with Hg alone, the lowest concentration of the extract, 5 µg/mL showed slight cytoprotective effect, with viability remaining at 76%. Conversely, significant cytoprotection was observed at 25 µg/mL, increasing cell viability to approximately 83%. The maximal protective effect was achieved at 50 µg/mL, where Momast restored cell viability to approximately 96%, a value approaching that of untreated control cells (Figure 3b). Exposure to 25 μM Pb resulted in a moderate but statistically significant reduction in cell viability (approximately 88% of the control value). Co-treatment with Momast significantly attenuated the Pb-induced decrease in cell viability (Figure 3c). Significant protection was observed at both 5 and 25 μg/mL, restoring cell viability to approximately 95% of the control value. At the highest concentration (50 μg/mL), Momast further increased cell viability to approximately 105% of the control value.

**Figure 4:**
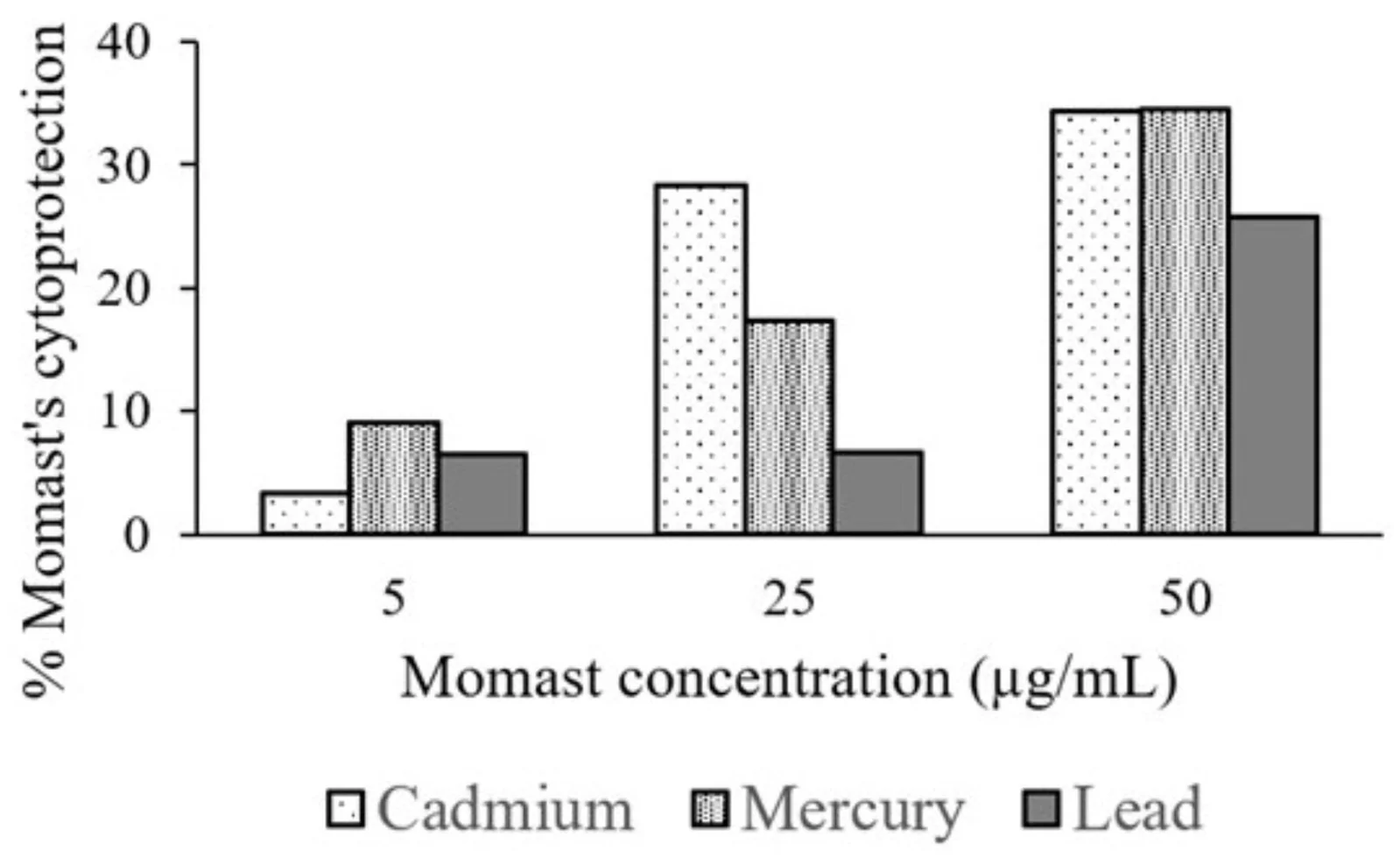
Percentage of Momast’s cytoprotection at different concentrations. Percentage values (Δ%) of cytoprotection of Momast (5, 25, and 50 µg/mL) in combination with CdCl_2_, HgCl_2_, and PbCl_2_ (25 µM) after 24 h of incubation. Δ% = [(Cell viability with heavy metal + Momast) − (Cell Viability with heavy metal)]/(Cell viability with heavy metal).

To facilitate comparison among the three experimental conditions, the effect of Momast was expressed as the relative increase in cell viability (Δ%) compared with the viability remaining after exposure to the corresponding heavy metal alone (Figure 4).

Under all conditions, Δ% increased with increasing Momast concentration, although the concentration–response profiles differed markedly among Cd-, Hg-, and Pb-treated cells. Distinct concentration–response profiles were observed depending on the heavy metal. In Cd-treated cells, Δ% increased markedly between 5 and 25 μg/mL, followed by a smaller increment at 50 μg/mL. In Hg-treated cells, Δ% increased almost linearly across the concentration range tested. Conversely, Pb-treated cells exhibited only modest increases in Δ% at 5 and 25 μg/mL, whereas the largest increment was observed at 50 μg/mL. Nevertheless, the overall relative increase in cell viability achieved in Pb-treated cells remained lower than that observed under Cd- and Hg-induced toxicity.

### 2.4. Effect of Momast’s Different Concentrations on PLM Capacitance

POPC:Ch PLMs were employed to investigate Momast’s ability to incorporate into the membrane and form channel-like events.

Following Momast’s addition, changes in PLM capacitance were observed at all concentrations tested (Table 2).

At 6.675, 2.667, and 0.267 μg/mL, membrane capacitance progressively increased during the lag phase (the interval between Momast addition to the *cis* cell medium and the appearance of the first channel-like event) before reaching a stable plateau. In contrast, at 0.0267 and 0.00267 μg/mL, capacitance decreased during the lag phase and subsequently remained constant throughout the recording period. The lag time increased markedly as Momast concentration decreased, from approximately 30 min at the three highest concentrations to 5400 and 7200 s at 0.0267 and 0.00267 μg/mL, respectively.

### 2.5. Effect of Momast’s Different Concentrations on PLM Conductance

Across the five experimental sets, Momast’s ability to incorporate into PLM and form channel-like events varied depending on the Momast concentration.

**Figure 5:**
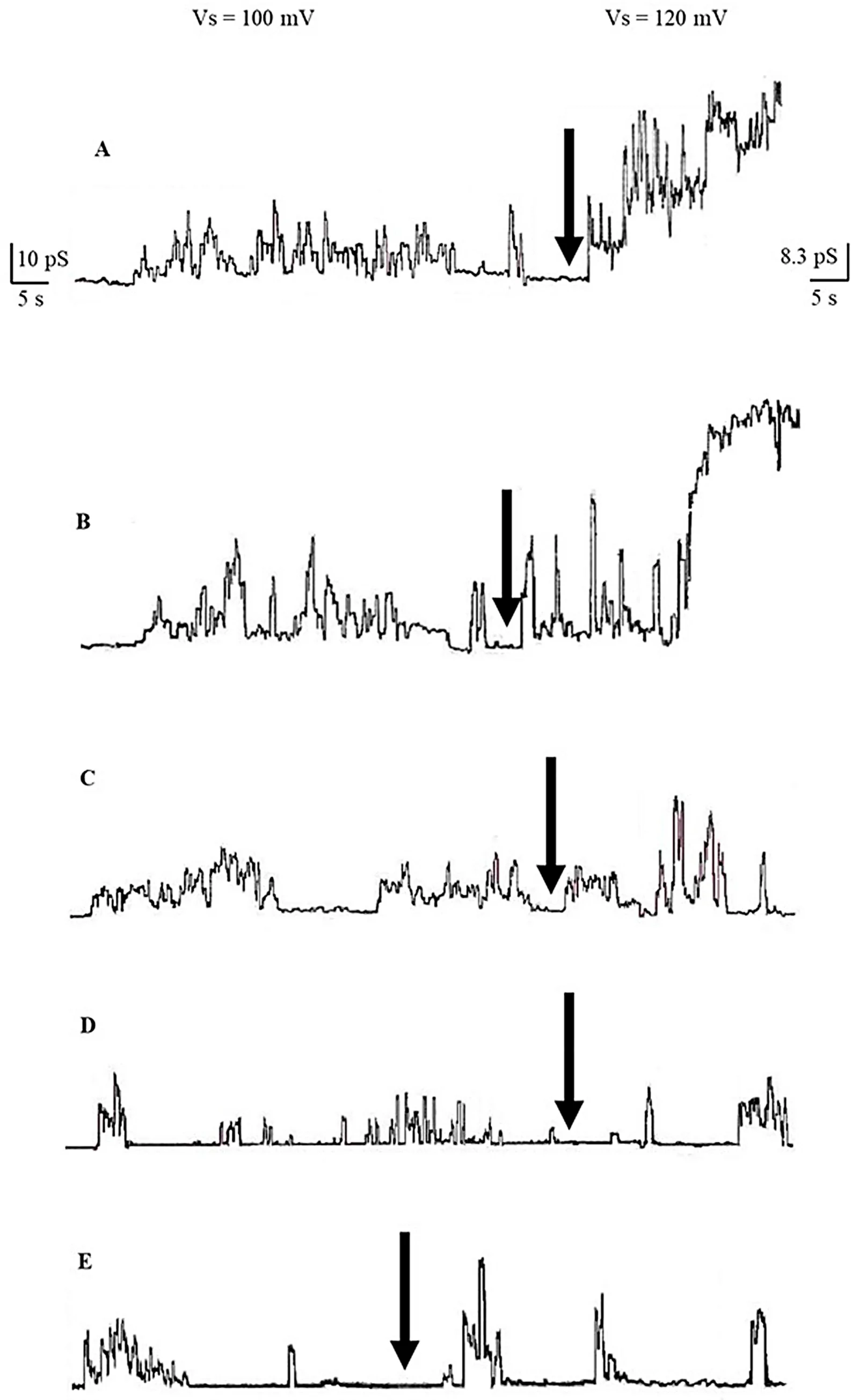
Momast channel-like activity in POPC:Ch PLMs. Example of chart recordings of Momast’s channel-like activity in PLMs made up of POPC:Ch. Black arrows indicate the change in applied voltage. (**A**,**B**) show two representative traces illustrating paroxystic activity. Experiments were performed in the presence of 6.675 (**A**), 2.667 (**B**), 0.2667 (**C**), 0.02667 (**D**) and 0.002667 (**E**) µg/mL of Momast added to the *cis* side, while the aqueous phase contained 1 M KCl (pH 7) and T = 23 ± 1 °C.

Channel-like activity was observed at all Momast concentrations, although its electrophysiological characteristics depended on both Momast concentration and applied voltage (Figure 5). At the three highest concentrations (6.675, 2.667, and 0.267 μg/mL), channel-like events appeared after a lag time of approximately 30–35 min and consisted of discrete multilevel conductive events alternating with quiescent periods. Channel activity was detected mainly at applied voltages between ±80 and ±120 mV, whereas no events were observed between ±20 and ±60 mV.

At 6.675 μg/mL, application of ±120 mV rapidly induced paroxysmal activity followed by membrane rupture. At 2.667 μg/mL, paroxysmal activity developed only after prolonged recording at ±120 mV, whereas at 0.267 μg/mL stable multilevel events were maintained throughout the recording period.

The electrophysiological parameters are summarized in Table 3 and Table 4. Central conductance (Λ_c_ ± SE) values were comparable among Momast concentrations at the same applied voltage, whereas event frequency (F ± SD) reached its highest values at 0.267 μg/mL.

### 2.6. Duration, Po, and Size of Momast-Induced Conductive Units in PLMs

Duration, Po, and size are additional biophysical parameters characterizing the conductive unit.

The duration and estimated diameter of Momast-induced conductive units were largely independent of Momast concentration, ranging between 0.75–1.25 s and 1.00–1.06 Å, respectively (Table 5). Likewise, central conductance remained unchanged across the concentration range investigated. In contrast, the open probability (Po) exhibited a concentration-dependent profile, reaching its maximum value at 0.267 μg/mL. This trend paralleled the increase in event frequency, indicating that this concentration produced the highest channel activity.

Overall, Momast induced the formation of conductive units over the entire concentration range investigated. While conductance, duration, and estimated pore diameter remained substantially unchanged, Po and event frequency reached their highest values at 0.267 μg/mL (Table 5).

## 3. Discussion

The present study investigated the potential protective effects of Momast against heavy metal-induced toxicity in SH-SY5Y neuroblastoma cells and explored whether its effects may involve direct interactions with the lipid component of biological membranes. Overall, the results indicate that Momast exerts a modest but significant cytoprotective effect against Cd-, Hg-, and Pb-induced toxicity, while the planar lipid membrane experiments provide evidence of a direct interaction with the lipid bilayer. Together, these findings support the involvement of complementary membrane-dependent and intracellular mechanisms.

The chemical characterization of Momast confirmed its high phenolic content and antioxidant capacity. The higher total phenolic content obtained with the Folin–Ciocalteu assay compared with the Fast Blue BB assay is consistent with the different chemical selectivity of the two methods, as the former can also respond to non-phenolic reducing compounds [36,37]. Nevertheless, both approaches confirmed the high phenolic content of the tested preparation. Moreover, the TPC values obtained in the present study were higher than those previously reported for commercially available Momast formulations, including HY100, PLUS30, PW25, and MP30B [15,16].

While the TPC values obtained in the present study were higher than those previously reported for specific commercial Momast matrix variations (such as HY100, PLUS30, PW25, or MP30B) [15,38], this reflects differences in formulation concentration grades rather than uncontrolled botanical source. Because Momast is a patented extract derived from a single regional cultivar (Coratina) using a standardized, solvent-free mechanical process, its main polyphenolic fingerprint remains strictly controlled via official IOC HPLC methods [39]. Furthermore, to account for minor natural matrix fluctuations and ensure analytical rigor, in this study, TPC was evaluated using two complementary assays (Folin–Ciocalteu and Fast Blue BB). This dual-testing approach, combined with industrial QA/QC standardization, ensures high batch-to-batch consistency and can support the reproducibility of the reported biological results. The antioxidant assays further indicated a marked reducing and radical-scavenging capacity, although the relative responses differed among ABTS, DPPH, and FRAP. Such differences are expected because these assays measure partially distinct aspects of antioxidant activity [40,41].

These findings provide a biochemical basis for investigating whether the antioxidant properties of Momast may contribute to its cellular effects.

In the present study, we investigated the neuroprotective potential of Momast using complementary in vitro models.

The three heavy metals produced clearly different toxicity profiles in SH-SY5Y cells, with cadmium showing the greatest cytotoxicity, followed by mercury and lead. This ranking is consistent with previous observations in SH-SY5Y cells [42] and may reflect differences in the cellular targets and mechanisms of toxicity of the three metals [43].

The concentrations tested in the present study (0.25–250 μM) were selected to encompass a broad range of exposure levels, from concentrations producing limited or no detectable effects to concentrations causing marked loss of cell viability. This approach allowed the relative sensitivity of SH-SY5Y cells to the three metals to be characterized and provided a rationale for selecting 25 μM as a common concentration for the subsequent cytoprotection experiments.

The distinct toxicity profiles of Cd, Hg, and Pb can be related to their different molecular targets. Cadmium readily perturbs cellular redox homeostasis and can impair mitochondrial function, calcium and zinc signaling, endoplasmic reticulum homeostasis, and antioxidant defenses, thereby promoting oxidative stress and cell death. Mercury has a high affinity for sulfhydryl groups and can directly interfere with thiol-containing proteins, mitochondrial function, and redox homeostasis, leading to oxidative and membrane-associated damage. Lead, in contrast, can mimic or interfere with calcium-dependent signaling and disrupt neurotransmission, neuronal differentiation, and intracellular signaling pathways, with oxidative stress contributing to its downstream effects [43,44,45]. These differences provide a biological rationale for the different concentration–response profiles observed in our experiments and for the greater sensitivity of SH-SY5Y cells to Cd and Hg compared with Pb. Importantly, the concentrations producing measurable effects in vitro should not be interpreted as direct equivalents of human exposure levels, but rather as experimentally relevant concentrations for resolving metal-specific cellular responses.

Momast attenuated the reduction in cell viability induced by all three metals, although the magnitude and concentration dependence of this effect differed according to the metal. The protection against Pb-induced toxicity was evident mainly at the highest Momast concentration, whereas Cd-induced toxicity showed the most pronounced response at an intermediate concentration, and the protective effect against Hg increased progressively with Momast concentration. These different responses suggest that Momast does not act through a single mechanism but may interfere with multiple cellular processes affected by each metal. The relatively higher Momast concentration required to counteract Pb toxicity may be related to the predominant involvement of calcium-dependent signaling and neuronal dysfunction in lead toxicity, whereas the stronger effects observed against Cd and Hg may be more directly influenced by Momast antioxidant and membrane-associated properties.

The PLM experiments were performed to determine whether Momast can interact directly with a lipid bilayer independently of cellular proteins and intracellular signaling pathways. Planar lipid membranes composed of POPC and 35 mol% cholesterol (POPC:Ch = 65:35) represent a simplified biomimetic model of the outer leaflet of aged neuronal plasma membranes in which changes in membrane capacitance and electrical conductance could be monitored under controlled conditions.

Capacitance relates to a membrane’s ability to function as a capacitor and is inversely proportional to its thickness. The capacitance parameter is considered the most reliable indicator of the stability and structural integrity of the lipid bilayer before and after experiments involving the incorporation of other substances [46].

The observed capacitance changes were consistent with a sequential process involving initial adsorption of Momast components at the membrane surface followed by their penetration into the hydrophobic region of the bilayer, without evidence of irreversible membrane destabilization. This observation is relevant because individual Momast phenolic constituents, including hydroxytyrosol, verbascoside, and oleuropein-related compounds, have previously been shown to interact with or cross lipid membranes [47,48,49,50].

At the same time, the appearance of discrete conductance events indicates that Momast can induce transient conductive assemblies within the lipid bilayer. The conductance, frequency, and open probability of these events suggest the formation of organized structures capable of allowing ionic flux. Although the available data do not allow the precise molecular architecture of these assemblies to be established, their electrical characteristics are compatible with the formation of membrane-associated conductive structures, as previously proposed for polyphenol–lipid interactions [51]. Importantly, Momast concentrations used in the PLM experiments were substantially lower than those used in the cellular assays because the electrophysiological approach is considerably more sensitive and higher concentrations destabilized the lipid membranes. Therefore, the different concentration ranges used in the two experimental systems reflect their different analytical sensitivity rather than a direct comparison of biological doses.

The combined findings support, but do not by themselves establish, a model in which Momast may exert cytoprotective effects through complementary membrane-associated and intracellular mechanisms. Direct interaction with the lipid bilayer could contribute to the preservation of membrane properties and potentially limit membrane-associated oxidative damage, while the ability of Momast components to associate with and transiently permeate the membrane may facilitate their intracellular availability. Once inside the cell, these compounds could contribute to the modulation of oxidative and other metal-induced cellular responses. This interpretation remains mechanistic and requires further investigation, but it provides a plausible framework linking the antioxidant properties of Momast, its direct interaction with lipid membranes, and its protective effects in SH-SY5Y cells.

In conclusion, the present findings indicate that Momast can partially counteract heavy metal-induced cytotoxicity in a neuronal cell model and that this effect is accompanied by a direct interaction with lipid membranes. The metal-dependent differences in cytoprotection, together with the PLM results, suggest that both membrane-associated and intracellular processes contribute to the observed effects. Further studies using specific molecular markers and cellular uptake approaches will be necessary to define the relative contribution of these mechanisms.

## 4. Materials and Methods

### 4.1. Chemicals

1-palmitoyl-2-oleoyl-sn-glycero-3-phosphocholine (POPC) was purchased from Avanti Polar Lipids (Alabaster, AL, USA). Cholesterol, potassium chloride (KCl), cadmium chloride (CdCl_2_), mercury chloride (HgCl_2_), lead chloride (PbCl_2_), and n-decane were obtained from Sigma-Aldrich (St. Louis, MO, USA). MOMAST^®^ (Momast) was kindly provided by Bioenutra S.r.l. (Ginosa, TA, Italy) as a stock solution (32.068 mg/mL). Momast is a patented polyphenolic complex obtained through a standardized, physical and mechanical filtration process of vegetation water derived from Apulian olive pressing (*Olea europaea* L., predominantly Cultivar Coratina) [15,38]. Its phenolic composition (characterized primarily by hydroxytyrosol, tyrosol, verbascoside, and oleuropein derivatives) is verified by the company by using HPLC-DAD in accordance with the International Olive Council official method (IOC/T.20/Doc. No. 29) [14,39].

### 4.2. Total Phenolic Content by the Folin–Ciocalteu Assay

The total phenolic content (TPC) of Momast was determined using the Folin–Ciocalteu method as previously described by Di Salvo et al. [52], with measurements performed in 96-well microplates using an Infinite Pro 200 microplate reader (Tecan, Männedorf, Switzerland). Gallic acid (0.025–0.20 mg/mL) was used for calibration (y = 2.8194x + 0.0208, R^2^ = 0.9908). Briefly, 13 μL of Momast or gallic acid standard solution was mixed with 13 μL methanol, 13 μL Folin–Ciocalteu reagent, and 50 μL distilled water. After 5 min, 50 μL of 12% Na_2_CO_3_ and 113 μL of distilled water were added. Following incubation for 90 min at 30 °C in the dark, absorbance was measured at 710 nm. Results were expressed as milligrams of gallic acid equivalents per milliliter of Momast (mg GAE/mL).

### 4.3. Total Phenolic Content by the Fast Blue BB Assay

The TPC was also determined using the Fast Blue BB assay according to Di Salvo et al. [52]. A 0.1% Fast Blue BB solution was freshly prepared in distilled water. Gallic acid (0.01–0.28 mg/mL) was used as the calibration standard (y = 3.788x + 0.0229, R^2^ = 0.9976). Briefly, 100 μL of Fast Blue BB reagent was added to 1 mL of Momast or standard solution and mixed for 1 min. Subsequently, 100 μL of 5% NaOH was added, and the mixtures were incubated in the dark for 90 min at room temperature. Aliquots (200 μL) were transferred to 96-well plates and absorbance was recorded at 420 nm. Results were expressed as mg GAE/mL.

### 4.4. DPPH Radical Scavenging Assay

The antioxidant activity of Momast was evaluated using the DPPH assay according to Casciaro et al. [53]. Trolox (0.0025–0.15 mg/mL) was used as the reference antioxidant (R^2^ = 0.9943). Briefly, 20 μL of Momast or Trolox standard was mixed with 180 μL of DPPH solution (0.05915 mg/mL) prepared in methanol/water (80:20, *v*/*v*). Following incubation for 40 min at room temperature in the dark, absorbance was measured at 515 nm.

Radical scavenging activity was calculated as:

(1) $$\%\mathrm{DPPH}\;\mathrm{inhibition}=\left[1-\frac{A_{sample}-A_{blank}}{A_{control}-A_{blank}}\right]\times 100$$

where A_sample_, A_blank_ and A_control_ represent the absorbance of the sample, blank and control, respectively. Antioxidant activity was expressed as milligrams of Trolox equivalents per milliliter of Momast (mg TE/mL).

### 4.5. ABTS Radical Cation Scavenging Assay

The antioxidant capacity of Momast was also evaluated using the ABTS assay following the method described by Di Salvo et al. [52]. The ABTS^+^ working solution was prepared by reacting 7 mM ABTS with 2.45 mM potassium persulfate for 16 h in the dark and subsequently diluted with methanol to an absorbance of 0.74 ± 0.03 at 734 nm. Trolox (0.0025–0.15 mg/mL) served as the calibration standard (R^2^ = 0.9940). Ten microliters of Momast or Trolox solution were mixed with 190 μL of ABTS working solution in 96-well plates. After incubation for 10 min at room temperature in the dark, absorbance was measured at 734 nm. Results were expressed as mg TE/mL.

### 4.6. Ferric Reducing Antioxidant Power (FRAP) Assay

The ferric reducing antioxidant power of Momast was determined according to Di Salvo et al. [52], with minor modifications. The FRAP working solution consisted of acetate buffer (300 mM), 10 mM TPTZ in HCl, and 20 mM FeCl_3_ mixed in a 10:1:1 ratio and incubated at 37 °C for 30 min before use. Trolox (0.0075–0.15 mg/mL) was used as the calibration standard (R^2^ = 0.9913). Briefly, 6 μL of Momast or Trolox standard solution was mixed with 194 μL of FRAP reagent and incubated at 37 °C for 15 min in the dark. Absorbance was then measured at 593 nm. Results were expressed as mg TE/mL.

### 4.7. Culture Cells

Preliminarily, stock solutions (100 mM) of CdCl_2_, HgCl_2_ and PbCl_2_ were prepared in sterile bidistilled water, filtered through 0.22 μm filters, stored at 4 °C, and diluted in culture medium immediately before use to obtain the desired final concentrations (0.25–250 μM).

SH-SY5Y human neuroblastoma cells were cultured in high-glucose DMEM supplemented with 10% fetal bovine serum, 4 mM L-glutamine, and 1% penicillin–streptomycin at 37 °C in a humidified atmosphere containing 5% CO_2_.

Cells were seeded into 96-well plates at 5 × 10^3^ cells/well and allowed to adhere for 24 h before treatment.

Cells were exposed for 24 h to:

- CdCl_2_, HgCl_2_ or PbCl_2_ (0.25–250 μM);
- Momast (5–50 μg/mL);
- CdCl_2_, HgCl_2_ or PbCl_2_ (25 μM) in combination with Momast (5–50 μg/mL).

Cell viability was evaluated using the MTT assay as previously described [54].

In brief, SH-SY5Y cells were seeded at a density of 5 × 10^3^ in a 96-well plate and then incubated with different concentrations of CdCl_2_, HgCl_2_, or PbCl_2_ and Momast for 24 h. After this time, the medium was removed from the well and incubated with 20 μL of MTT stock solution (5 mg/mL in PBS 1X) in 180 μL of medium for 3 h at 37 ◦C in the dark. The medium was removed, and 150 μL of DMSO was added to dissolve the formazan crystals and maintained under stirring for 5 min at room temperature. Finally, absorbance was recorded at 540 nm with a multilabel microplate reader Victor 3 (PerkinElmer, Waltham, MA, USA). All MTT assays were performed in triplicate. Cell viability was expressed as a percentage of the control group (% control) calculated from the equation:

(2) $$\%Control=\frac{Absorbance\;treatment}{Absorbance\;control}\times 100$$

Data were expressed as the mean percentages of viable cells vs. the respective controls.

Relative increase in cell viability (Δ%) induced by Momast compared with the corresponding heavy-metal-treated group was calculated according to the following equation:

(3) $$\Delta \%=\frac{\left({Cell\;viability}_{heavy\;metal+Momast})-({Cell\;Viability}_{withh\;eavy\;metal}\right)}{{Cell\;viability}_{with\;heavy\;metal}}\times 100$$

### 4.8. Single-Channel Measurement

Channel-like activity was observed in planar lipid membranes (PLMs) composed of POPC:Ch (65:35, *w*/*w*) in 1% n-decane. Bilayers were formed across a 300 μm aperture in the Teflon partition separating two 4000 μL Teflon chambers containing symmetrical 1 M KCl solutions (pH 7) at 23 ± 1 °C. Unbuffered aqueous solutions were used, and all salts were of analytical grade. Momast solutions at concentrations of 3206.8, 320.68, 32.068, and 3.2068 µg/mL were prepared from a 32,068 µg/mL stock solution through serial dilution. Different volumes (8.32 and 3.33 µL) of these solutions were added to the *cis* chamber and stirred to achieve final concentrations of 6.675, 2.667, 0.267, 0.0267, and 0.00267 µg/mL, respectively. Momast was added to the *cis* side of the medium facing the membrane at an applied voltage of 100 mV (addition voltage), regardless of the final Momast concentration.

The membrane current was monitored using an oscilloscope and recorded on a chart recorder for subsequent analysis. The *cis* and *trans* chambers were connected to the amplifier headstage via Ag/AgCl electrodes in series with a voltage source and a high-sensitivity current amplifier (OPA 129). The single-channel instrumentation had a time resolution of 1–10 ms, depending on the magnitude of the channel-like event conductance. Voltage polarity was defined with respect to the side where Momast was added (the *cis* side). A *trans*-negative potential (indicated by a minus sign) signifies that a negative potential was applied to the *trans* side, the chamber opposite to where Momast was introduced. These experiments are considered a sensitive technique for investigating channel-forming substances [55,56].

Electrophysiological analysis included determination of conductance, capacitance, frequency, open probability and pore diameter.

*Conductance Determination*: Conductance was determined by measuring the amplitude of channel-like events. Channel-like event data, filtered at 300 Hz, were obtained from at least three independent experiments (more than 130 channel-like events in total) performed on different days. A histogram of the conductance amplitude distribution was constructed and fitted with a Gaussian distribution function (GraphPad PrismTM version 3.0). Results are expressed as central conductance ± standard error (Λ_c_ ± SE).

*PLM Capacitance Measurement*: To determine the PLM capacitance, we used a calibration curve generated by simulating membrane capacitance with a discrete set of known capacitances (C_n_) and measuring the corresponding output voltage (*V_lh_*). The obtained data were fitted using the following formula:

(4) $$V_{lh}=\frac{A\times B}{\left(B+C_{n}\right)}$$

where A and B are free parameters derived from the fitting procedure. Their values were then used to convert *V_lh_* values into capacitance data.

*Frequency of Channel-like Events*: To determine the frequency (number of channel-like events per 60 s), any detection of channel-like events was counted as successful. Results were expressed as frequency ± standard deviation (F ± SD).

*Estimation of Momast Channel-like Event Size*: The size of the Momast channel-like event was estimated using the following formula:

(5) $$\Lambda_{c}=\frac{\left(\sigma \times \pi \times r^{2}\right)}{d}$$

where Λ_c_ is the central conductance, σ is the specific conductivity of the solution filling the channel, π has the value of 3.14, r is the channel-like event radius, and d is its length.

*Open Probability*: The open probability, Po, representing the fraction of time the channel-like event spends in the conductive state, was calculated as the ratio of the number of data points in the conductive levels to the total number of points in the recording [57,58].

### 4.9. Statistical Analysis

Data are presented as mean ± SD unless otherwise specified. To ensure reproducibility, all experiments were independently repeated at least three times, and each assay was performed in triplicate.

Cell viability data were analyzed using one-way ANOVA followed by Dunnett’s multiple comparison test, performed in GraphPad Prism 9.0.

Conductance data were analyzed by one-way ANOVA followed by Tukey’s multiple comparison test, performed using GraphPad Prism 3 software (GraphPad PrismTM version 3.0).

A value of *p* < 0.05 was considered statistically significant.

## Figures and Tables

**Table 1:** Total Phenolic Content (TPC) and Total Antioxidant Activity (TAA) results of the Momast sample.

	TPC		TAA		
	F-C	FBBB	DPPH	FRAP	ABTS
Momast	mgGAE/mL ± SD	mgGAE/mL ± SD	mgTE/mL ± SD	mgTE/mL ± SD	mgTE/mL ± SD
	209.07 ± 8.78	50.29 ± 0.57	247.66 ± 0.6	372.24 ± 3.01	244.96 ± 4.05

GAE = gallic acid equivalent; TE = Trolox equivalent. SD = standard deviation (n = 3).

**Table 2:** Capacitance variation in POPC:Ch PLM. Mean values of the membrane capacitance calculated just before the addition of Momast (Ci ± SE) and at the end of the experiment (Cf ± SE). The mean value was obtained from at least three experiments.

[Momast] µg/mL	Ci ± SE µF/cm^2^	Cf ± SE µF/cm^2^
6.675	0.35 ± 0.01	0.51 ± 0.02
2.667	0.32 ± 0.02	0.45 ± 0.01
0.267	0.32 ± 0.02	0.69 ± 0.03
0.0267	0.34 ± 0.10	0.20 ± 0.01
0.00267	0.32 ± 0.09	0.11 ± 0.02

SE = Standard Error.

**Table 3:** The mean conductance (Λ_c_ ± SE) of Momast conductive units at different applied voltages. The minimum and maximum number of conductive units considered (N) out of a total number of conductive units considered (Nt) was: [Momast]_f_ = 6.675 µg/mL, 169 < N < 384, Nt = 865; [Momast]_f_ = 2.667 µg/mL, 160 < N < 555, Nt = 905; [Momast]_f_ = 0.267 µg/mL, 170 < N < 388, Nt = 1242; [Momast]_f_ = 0.0267 µg/mL, 150 < N < 200, Nt = 900; [Momast]_f_ = 0.00267 µg/mL, 145 < N < 170, Nt = 620.

	[Momast] 6.675 µg/mL	[Momast] 2.667 µg/mL	[Momast] 0.267 µg/mL	[Momast] 0.0267 µg/mL	[Momast] 0.00267 µg/mL
Vs mV	Λ_c_ ± SE pS	Λ_c_ ± SE pS	Λ_c_ ± SE pS	Λ_c_ ± SE pS	Λ_c_ ± SE pS
120	-	14.6 ± 1.1	17.1 ± 1.2	15.9 ± 1.4	21.7 ± 3.4
100	19.7 ± 0.9	17.7 ± 1.4	19.9 ± 1.0	19.0 ± 1.0	17.9 ± 1.3
80	29.3 ± 1.4	26.0 ± 1.5	24.1 ± 0.8	24.8 ± 1.3	-
−80	28.3 ± 1.2	28.9 ± 1.4	25.7 ± 0.9	25.5 ± 1.6	-
−100	23.0 ± 1.3	20.4 ± 1.1	20.0 ± 0.9	19.9 ± 1.0	19.0 ± 1.6
−120	-	16.7 ± 1.2	15.5 ± 2.6	16.0 ± 0.8	15.7 ± 1.8

SE = Standard Error.

**Table 4:** The frequency (F ± SD) of Momast conductive units at different applied voltages. The minimum and maximum number of conductive units considered (N) out of a total number of conductive units (Nt) is reported in the legend in Table 3.

	[Momast] 6.675 µg/mL	[Momast] 2.667 µg/mL	[Momast] 0.267 µg/mL	[Momast] 0.0267 µg/mL	[Momast] 0.00267 µg/mL
Vs mV	F ± SD	F ± SD	F ± SD	F ± SD	F ± SD
120	-	3.1 ± 0.9	16.0 ± 1.7	6.5 ± 1.0	4.5 ± 0.7
100	9.5 ± 1.5	12.3 ± 2.3	15.8 ± 1.8	4.6 ± 0.7	2.3 ± 0.8
80	10.0 ± 1.4	11.3 ± 2.1	17.7 ± 0.9	2.8 ± 0.6	-
−80	11.2 ± 1.6	13.6 ± 1.9	19.2 ± 1.2	3.5 ± 0.5	-
−100	9.2 ± 1.5	11.1 ± 3.5	14.9 ± 0.9	3.6 ± 0.3	1.9 ± 0.7
−120	-	2.3 ± 0.2	9.4 ± 0.7	5.8 ± 0.5	3.9 ± 0.3

SD = Standard Deviation.

**Table 5:** Biophysical parameters characterizing the Momast conductive units. Duration, open probability (Po), and diameter of the conductive unit were calculated at different Momast concentrations, at applied voltages of ±100 mV. The minimum and maximum number of conductive units considered (N) out of a total number of conductive units considered (Nt) was: [Momast]_f_ = 6.675 µg/mL, 162 < N < 164, Nt = 326; [Momast]_f_ = 2.667 µg/mL, 160 < N < 172, Nt = 332; [Momast]_f_ = 0.267 µg/mL, 180 < N < 190, Nt = 370; [Momast]_f_ = 0.0267 µg/mL, 150 < N < 162, Nt = 312; [MP]_f_ = 0.00267 µg/mL, 151 < N < 159, Nt = 310.

	Vs = 100 mV			Vs = −100 mV		
[Momast] µg/mL	Duration s	Po	Diameter Å	Duration s	Po	Diameter Å
6.675	0.75–1.25	0.25	1.06	0.75–1.25	0.26	1.14
2.667	0.75–1.25	0.33	1.00	0.75–1.25	0.32	1.08
0.2667	0.75–1.25	0.53	1.06	0.75–1.25	0.50	1.07
0.0267	0.75–1.25	0.15	1.04	0.75–1.25	0.12	1.06
0.00267	0.75–1.25	0.094	1.01	0.75–1.25	0.10	1.04

## Data Availability

The data are contained within this article.

## Supplementary Materials

The following supporting information can be downloaded at: https://www.mdpi.com/article/10.3390/ijms27188157/s1.
