# Supplementary material for: Momast Protects SH-SY5Y Cells Against Heavy Metal-Induced Neurotoxicity Through Complementary Membrane-Dependent and Intracellular Mechanisms
Source: Int J Mol Sci. 2026 Sep 13;27(18):8157. doi: 10.3390/ijms27188157 (PMC13607061; doi:10.3390/ijms27188157)
Supplement: Supplementary file 1 [file ijms-27-08157-s001.zip › ijms-4490400-supplementary.pdf]

Supporting Material

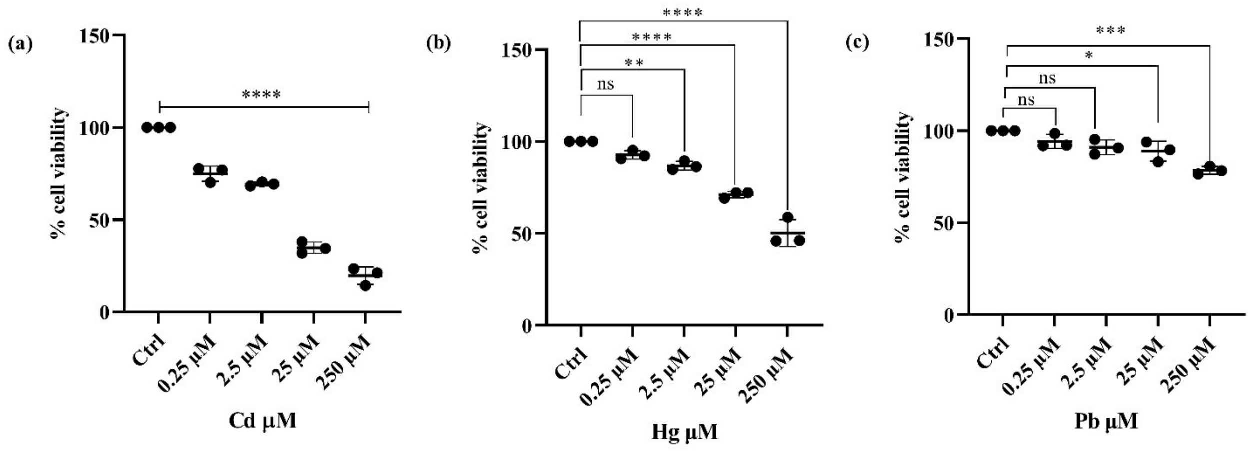

Figure S1. Effect of Cd (a), Hg (b), or Pb (c) on SH-SY5Y cell viability after 24 h of exposure (MTT assay). Data are expressed as a percentage of vehicle-treated cells (control). Individual data points represent three independent experiments, and results are shown as mean ± standard deviation (SD) (n = 3). Non-significant differences (ns, $P > 0.05$), * $P < 0.05$, ** $P < 0.01$, *** $P < 0.001$ and, **** $P < 0.0001$.

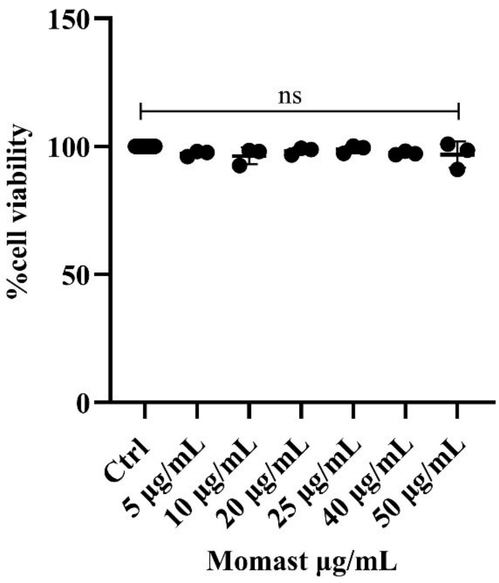

Figure S2. Effect of Momast on SH-SY5Y cell viability after 24 h of exposure (MTT assay). Data are expressed as a percentage of vehicle-treated cells (control). Individual data points represent three independent experiments, and results are shown as mean ± standard deviation (SD) (n = 3). No statistically significant differences were observed (ns, $P > 0.05$).

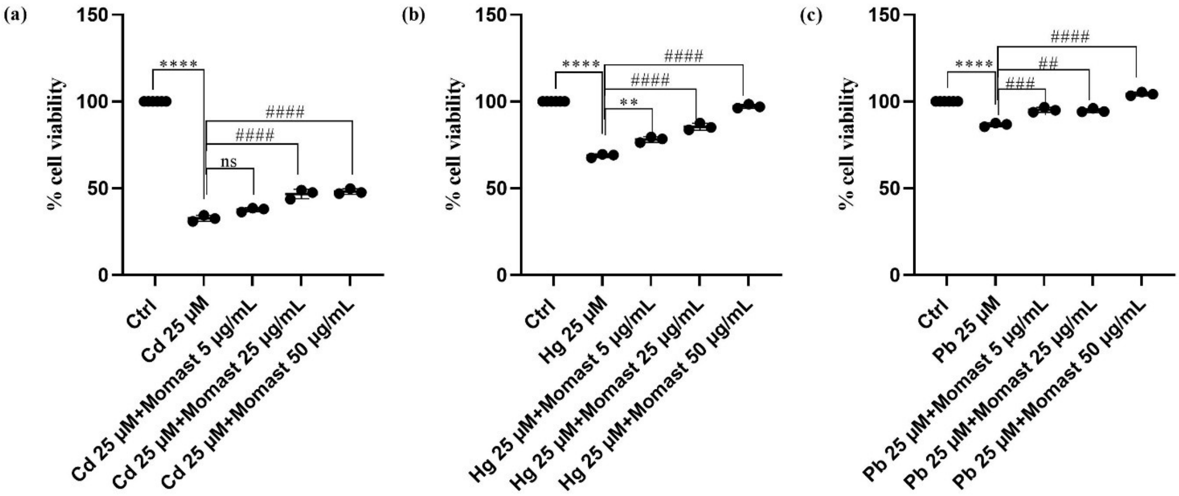

Figure S3. Effect of simultaneous treatment with Cd (a), Hg (b), or Pb (c) and Momast on SH-SY5Y cell viability after 24 h of exposure (MTT assay). Cells were treated with each heavy metal at 25 μM, either alone or in combination with Momast at 5, 25, or 50 μg/mL. Data are expressed as a percentage of vehicle-treated cells (control). Individual data points represent three independent experiments, and results are shown as mean ± standard deviation (SD) (n = 3). Asterisks indicate significant differences compared with the control, whereas hash symbols indicate significant differences compared with the corresponding heavy metal-treated group. Non-significant differences (ns, $P > 0.05$), ** $P < 0.01$, **** $P < 0.0001$, ## $P < 0.01$, ### $P < 0.001$, and #### $P < 0.0001$.

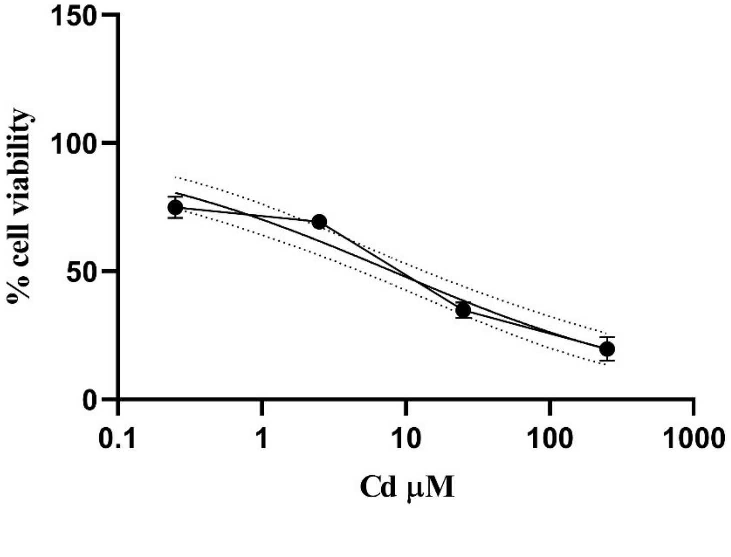

Figure S4. MTT dose–response curve of SH-SY5Y cells exposed to increasing concentrations of $CdCl_2$ for 24 h. Cell viability is expressed as a percentage of vehicle-treated cells (control). Results are shown as mean ± standard deviation (SD) from three independent experiments (n = 3). The concentration–response curve was obtained by nonlinear regression using a normalized variable-slope inhibition model. The calculated IC_{50} was 8.06 μM (95% CI: 4.85–13.32 μM; $R^2 = 0.9368$).

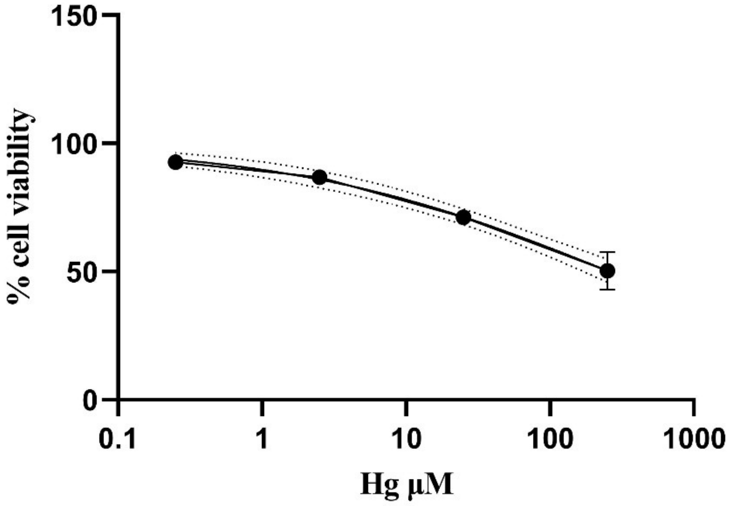

Figure S5. MTT dose–response curve of SH-SY5Y cells exposed to increasing concentrations of $HgCl_2$ for 24 h. Cell viability is expressed as a percentage of vehicle-treated cells (control). Results are shown as mean ± standard deviation (SD) from three independent experiments (n = 3). The concentration–response curve was obtained by nonlinear regression using a normalized variable-slope inhibition model. The calculated IC_{50} was 259.2 µM (95% CI: 170.6–444.9 µM; R^2 = 0.9582), close to the upper limit of the tested concentration range.

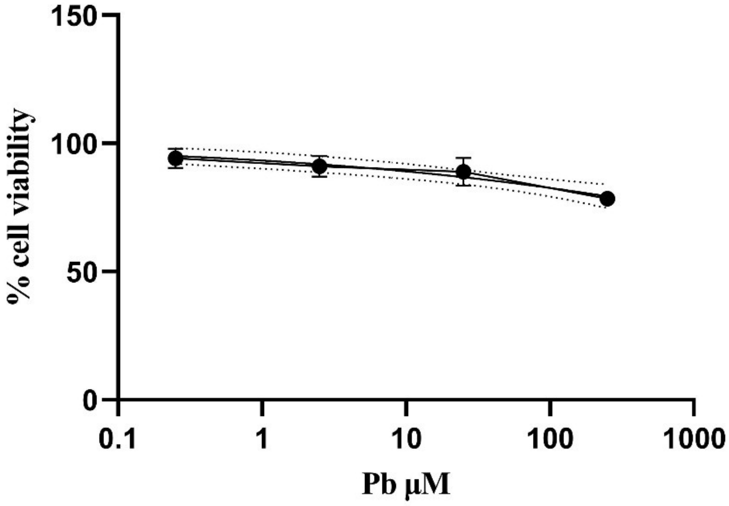

Figure S6. For $PbCl_2$, 50% inhibition of cell viability was not reached within the tested concentration range; therefore, the IC_{50} could not be reliably determined.

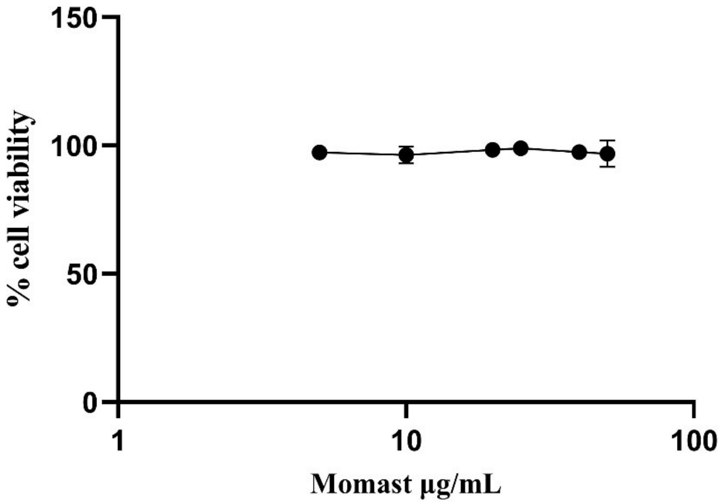

Figure S7. Concentration–response profile of SH-SY5Y cells exposed to increasing concentrations of Momast for 24 h. Cell viability was evaluated using the MTT assay and expressed as a percentage of vehicle-treated cells (control). Results are shown as mean ± standard deviation (SD) from three independent experiments (n = 3). Momast did not induce a concentration-dependent reduction in cell viability within the tested concentration range (5–50 µg/mL); therefore, an IC_{50} value could not be determined.
